# Multispectral Mid-Infrared Camera System for Accurate Stand-Off Temperature and Column Density Measurements on Flames

**DOI:** 10.3390/s21248395

**Published:** 2021-12-16

**Authors:** Juan Meléndez, Guillermo Guarnizo

**Affiliations:** LIR—Infrared Laboratory, Department of Physics, Universidad Carlos III de Madrid, 28911 Leganés, Spain; guillermoandres.guarnizo@uc3m.es

**Keywords:** infrared imaging, multispectral imaging, hyperspectral imaging, combustion monitoring, remote sensing and sensors, spectroscopy, fourier transform, image processing

## Abstract

Accurate measurement of temperature in flames is a challenging problem that has been successfully addressed by hyperspectral imaging. This technique is able to provide maps of not only temperature T (K) but also of column density Q (ppm·m) of the main chemical species. Industrial applications, however, require cheaper instrumentation and faster and simpler data analysis. In this work, the feasibility and performance of multispectral imaging for the retrieval of T and Q${}_{{CO}_{2}}$ in flames are studied. Both the hyperspectral and multispectral measurement methods are described and applied to a standard flame, with known T and Q${}_{{CO}_{2}}$, and to an ordinary Bunsen flame. Hyperspectral results, based on emission spectra with 0.5 cm${}^{-1}$ resolution, were found in previous works to be highly accurate, and are thus considered as the ground truth to compare with multispectral measurements of a mid-IR camera (3 to 5 μm) with a six interference filter wheel. Maps of T and Q obtained by both methods show that, for regions with T ≳1300 K, the average of relative errors in multispectral measurements is ∼5% for T (and can be reduced to ∼2.5% with a correction based on a linear regression) and ∼20% for Q. Results obtained with four filters are very similar; results with two filters are also similar for T but worse for Q.

## 1. Introduction

Combustion processes are of paramount importance in many economic sectors. Since temperature is the key factor that determines chemical reaction rates, an accurate control of it is essential to optimize these processes: monitoring of temperature improves consistency and energy efficiency in manufacturing, reduces wastage and pollution, increases reliability, and extends intervals of maintenance tasks. However, to measure physical parameters in a combustion process is a challenging problem, because intrusive probes can be damaged by the harsh environment, and even the toughest ones, such as thermocouples, induce perturbations in the flame, and provide readings with important systematic errors [1]. Therefore, non-intrusive methods, in particular those of optical thermometry, have become the state-of-the-art.

Active laser-based techniques, such as CARS (Coherent Anti-Stokes Raman Spectroscopy) or LIGS (Laser Induced Grating Spectroscopy) require complex laboratory setups that are difficult to install and operate in industrial environments, but still have a relatively large uncertainty, generally not better than 5% [2]. A promising alternative is passive techniques such as gas emission spectroscopy, whose setups are simpler since no excitation of the flame is required. In particular, the main chemical species in a flame have very specific emission profiles in the mid-IR band, that make possible their identification, quantization and, in principle, the measurement of their temperature.

One of the aims of the *European Metrology Programme for Innovation and Research* (*EMPIR*) project *EMPRESS* [2] (Enhancing process efficiency through improved temperature measurement) was to validate these spectroscopic techniques by measuring a standard flame of well-known temperature and chemical species concentration. The flame was developed by the British *National Physical Laboratory* (NPL), and its temperature was measured by Rayleigh scattering thermometry (a technique traceable to the International Temperature Scale of 1990, ITS-90) at NPL, and by Fourier Transform Infrared (FTIR) emission spectroscopy by two research groups, one at the *Technical University of Denmark* (DTU) and the other at *Carlos III University of Madrid* (UC3M). It was found that the agreement between all measurements amounted to 1%, that is, ΔT∼20 K for flame temperatures ∼2000 K [3].

This is a very good result that demonstrates the accuracy of flame temperature measurement based on emission spectroscopy. The advent of portable Fourier-transform hyperspectral imagers in the mid-IR region (also called Imaging Fourier Transform Spectrometers, IFTS) makes it possible to apply this technique to obtain temperature maps of flames in-situ, without any ad-hoc setup, simply by acquiring and processing the spectral radiance emitted by the flame. An additional advantage of the method is that it provides also maps of concentrations for the main chemical species. Although, in contrast to temperature, we have no reliable independent measurements to compare with, results for the standard flame have shown an agreement with the calculations of the chemical equilibrium software *GasEq* [4] better than 15% for CO${}_{2}$ and better than 10% for CO [5].

However, hyperspectral imagers in the mid-IR are expensive, and data processing is complex and time-consuming. Practical application in industrial environments requires cheaper and simpler instrumentation and faster processing, even at the expense of a not-so-good accuracy. This target has been addressed in the second part of the referred *EMPIR* project, *EMPRESS 2* [6].

The basic approach is to use a mid-IR camera that becomes a multispectral instrument by using interference filters. In this low-cost instrument, the spectral radiance integrated over six bands provides the equivalent of a low-resolution emission spectrum of the flame, whose shape and intensity depend on the temperature T${}_{f}$ and column density Q${}_{f}$ (concentration × optical path) of the chemical species of the flame. Simulations of emission spectra for a whole range of values of temperature $\left\{T_{i}\right\}$ and column density $\left\{Q_{i}\right\}$ are made using a line-by-line model that extracts the spectral parameters from the HITEMP2010 spectral database [7]. These simulated spectra are then integrated over the spectral bands of the camera and the results are compared with the measured radiances in each pixel; the couple (T${}_{f}$, Q${}_{f}$), which gives the best agreement, provides the retrieved values of temperature and column density for the pixel.

This methodology overcomes the basic problem of thermography when applied to flames or, in general, gas plumes: the unknown emissivity of the target. This is also a problem in the thermography of solids, but it can be dealt with by assuming a smooth spectral dependence of emissivity, and using Bayesian or regularization methods to solve the coupling between temperature and emissivity effects in spectral radiance [8,9]. The spectral structure of gases, in contrast, is extremely complex, with thousands of discrete absorption-emission lines. For that reason, state-of-the-art flame pyrometry resorts to measuring soot emission, generally in at least two bands the visible range [10]. This approach, however, is limited to sooty flames. In contrast, resorting to simulated spectra effectively parametrizes spectral emissivity as a function of T and Q, so that, if measurements are restricted to a spectral region where emission is due to a single chemical species (in our case, CO${}_{2}$), values of radiance in a few bands (in principle even two), could determine not only temperature, but also chemical composition, for any flame.

The feasibility of the multispectral method was demonstrated in a previous work [11], where the uncertainty of this procedure was estimated theoretically by a Montecarlo method. It was found that, for the typical values of T and CO${}_{2}$ in the standard flame, expected errors due to radiance fluctuations were very small: ΔT∼11 K and $\Delta Q_{CO_{2}}∼35$ ppm·m. Errors for the first experimental results, however, were an order of magnitude larger, but no systematic analysis of the accuracy of the results was made, and only the standard flame in stoichiometric conditions was measured. In this paper, we present the full results achieved by the UC3M group within the *EMPRESS 2* project. The basic approach has been to study experimentally the accuracy of the results of the multispectral method by comparing them to those of the hyperspectral method in a variety of flames: the standard flame in stoichiometric, lean, and rich conditions, and an ordinary Bunsen flame. Since the hyperspectral method has demonstrated an excellent accuracy in the EMPRESS project, its results have been considered as a “ground truth” for temperatures, making it possible to calculate statistical benchmarks to qualitatively assess the accuracy of multispectral results. Column densities retrieved by both methods have been also compared, although in this case the accuracy of hyperspectral results has not been tested by independent measurements.

The effect of the number of filters has been studied, showing that temperature results with only two filters are nearly as good as those using six bands. The feasibility of bi-spectral measurement of flame temperatures opens the possibility of fast and cheap temperature imaging, with important industrial applications.

The structure of the paper is as follows. Section 2 explains the radiative model used and the fundamentals and implementation of the two measurement methods employed—hyperspectral and multispectral. Section 3 outlines the experimental setup and the instrumentation used, and Section 4 describes the experimental results using six filters, comparing multispectral and hyperspectral T and Q values both for the standard flame and the Bunsen flame. Multispectral results obtained with a reduced number of filters are studied in Section 5, showing that, even with only two filters, properly chosen, good results are achieved for temperature. Finally, Section 6 summarizes the conclusions and suggests future work.

## 2. Measurement Method: From Hyperspectral to Multispectral

### 2.1. Radiative Model

**Figure 1:**
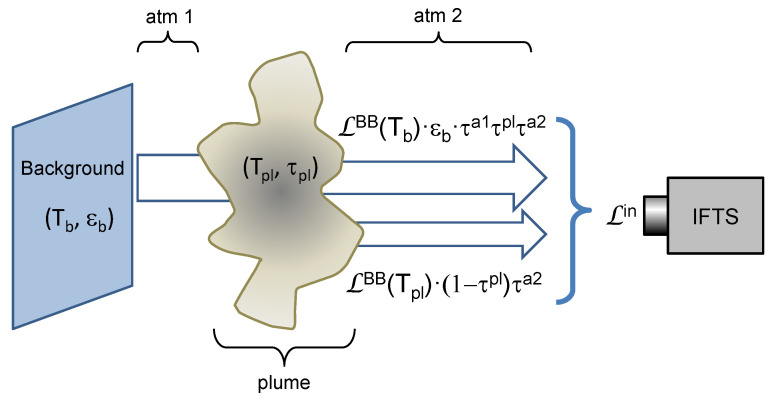
Schematics of the radiative model.

In a typical experimental setup for gas measurement with an IFTS (Figure 1), the instrument images a gas plume against a background, measuring the spectral radiance incoming to each pixel. In order to relate this radiance to the plume parameters, a radiative model of the measurement configuration is needed. In this work, we will follow the model explained in detail in [12]. For simplicity, the plume has been modeled by a single value of T and Q for each pixel; in a non-homogeneous flame, these values should be considered as line-of-sight averages.

It is assumed that atmospheric emission is negligible, the background emissivity $\varepsilon_{b}$ is large (so that reflection in the background is negligible), the gas is in local thermal equilibrium (so that Boltzmann distribution holds), and the effects of absorption and scattering by particulate matter are negligible. With these approximations the incoming radiance at the radiometer is:

(1) $$\begin{array}{c} L^{in}=L^{BB}\left(T_{b}\right)\;\cdot \;\varepsilon_{b}\;\cdot \;\tau^{a1}\tau^{pl}\tau^{a2}+L^{BB}\left(T_{pl}\right)\;\cdot \;\left(1-\tau^{pl}\right)\tau^{a2}, \end{array}$$

where $\tau^{pl}$, $\tau^{a1}$ and $\tau^{a2}$ are, respectively, the transmittances of the plume and the first and second atmospheric paths (atm 1 and atm 2 in Figure 1), $L^{BB}$ stands for Planck’s blackbody radiance, and $T_{b}$, $T_{pl}$ are the temperatures of background and plume.

Transmittances are given by Lambert–Beer’s law, that is, for a single chemical absorbing species, with an optical path *L*,

(2) $$\begin{array}{c} \tau \left(\nu ,C,T\right)=e^{-\alpha (\nu ,T)CL}\equiv e^{-\alpha (\nu ,T)Q}, \end{array}$$

where *C* is the concentration, Q≡CL is the column density (measured usually in parts per million per meter, ppm·m), and the dependence of the absorptivity α on wavenumber and temperature has been shown explicitly. Absorptivities are generally well known and can be extracted from spectroscopic databases like PNNL [13], HITRAN [14], or its high-temperature version, HITEMP2010 [7]. If there is more than one absorbing species, τ(ν) is just a product of terms like Equation (2), one for each species; if the concentration is not homogeneous, the product αCL is replaced by an integral.

Our aim is to obtain, from experimental measurements of $L^{in}\left(\nu \right)$, the values of plume temperature $T_{pl}$ and column density $Q_{pl}$ at each pixel, in order to have a “temperature map” and a “column density map” of the scene. It is not possible, however, to solve Equation (1) for $T_{pl}$ and $Q_{pl}$, because they are coupled in the Lambert–Beer expression of transmittance (Equation (2)), where the absorptivity α depends on $T_{pl}$ in a nontrivial way. Instead, our approach will be to calculate theoretical spectra as a function of $T_{pl}$ and $Q_{pl}$ and assign to each pixel the values that provide the best fit to its experimental spectrum.

#### Case of Very Hot or Very Cold Backgrounds

This process can be somewhat simplified for the extreme cases of very hot or very cold backgrounds. When the background is much hotter than the plume, the second term in Equation (1) can be neglected. In this *absorption* (or *active*) *mode*, Equation (1) becomes:

(3) $$\begin{array}{c} L_{abs}^{in}\approx L^{BB}\left(T_{b}\right)\;\cdot \;\varepsilon_{b}\;\cdot \;\tau^{a1}\tau^{pl}\tau^{a2}. \end{array}$$

The plume transmittance is then:

(4) $$\tau^{pl}\left(\nu ,Q_{pl},T_{pl}\right)\approx \frac{L_{abs}^{in}\left(\nu \right)}{L^{BB}\left(\nu ,T_{b}\right)\;\cdot \;\varepsilon_{b}\;\cdot \;\tau^{a}\left(\nu ,Q_{a},T_{a}\right)},$$

where $\tau^{a}=\tau^{a1}\;\cdot \;\tau^{a2}$ stands for the total atmospheric transmittance. If $T_{b}$, $\varepsilon_{b}$ and $\tau^{a}$ are known, $Q_{pl}$, and $T_{pl}$ can be determined by fitting the theoretical τ (Equation (2)) to this experimental value. Furthermore, the denominator in Equation (4) is just the reference spectrum $L_{ref}^{in}$ that can be measured experimentally if it is possible to turn off the plume, so that:

(5) $$\tau^{pl}\left(\nu ,Q_{pl},T_{pl}\right)\approx \frac{L_{abs}^{in}\left(\nu \right)}{L_{ref}^{in}\left(\nu \right)}.$$

Since transmittance is measured as a ratio, in *absorption spectroscopy* a radiometric calibration of the instrument is not necessary, as long as it has a linear response.

If, on the other hand, the background is much cooler than the plume, its contribution to the measured radiance will be negligible. This is the *emission* (or *passive*) *mode*. In this case, Equation (1) becomes:

(6) $$L_{emi}^{in}\left(\nu \right)\approx L^{BB}\left(T_{pl}\right)\;\cdot \;\left(1-\tau^{pl}\right)\;\cdot \;\tau^{a2}$$

and the experimental transmittance spectrum is:

(7) $$\tau^{pl}\left(\nu ,Q_{pl},T_{pl}\right)\approx 1-\frac{L_{emi}^{in}\left(\nu \right)}{L^{BB}\left(\nu ,T_{pl}\right)\;\cdot \;\tau^{a2}\left(\nu ,Q_{a2},T_{a2}\right)}.$$

This is formally similar to Equation (4), but there is a crucial difference. Whereas the denominator in Equation (4) can be easily estimated, or measured with a reference spectrum, in Equation (7) it contains the plume temperature, which is precisely what has to be determined. So, transmittance cannot be measured experimentally with this simple setup, and the approach of *emission spectroscopy* is to fit the measured radiance by simulated spectra, calculated with the right-hand side of Equation (6). This means that the radiometer must be accurately calibrated in radiance.

Measurements of flames, like those performed in this work, will nearly always be correctly modeled by this emission approximation.

### 2.2. Measurement Process: Hyperspectral

The process to obtain accurate temperature values from a flame through the hyperspectral imaging method can be described in three stages: acquisition of experimental spectra, calculation of simulated spectra, and a comparison of both.

The procedure to obtain experimental spectra begins with the output of the IFTS, which is a datacube that contains one interferogram for each pixel of the scene. Each interferogram must be Fourier-transformed to become the emission spectrum, but several additional processing steps must be performed in order to optimize the result [12]: correction of the DC component to compensate for signal drifts, apodization to improve lineshapes, zero padding to optimally interpolate spectra, phase correction to take into account small asymmetries in the interferogram, and off-axis correction to account for the effect of the distance from the optical axis on the interferograms of pixels away from the image center. In the IFTS used in this work, an additional correction has to be made to compensate for the non-symmetrical scan of the moving mirror in the Michelson interferometer for high-resolution spectra [15]. Finally, in flame measurements low-frequency fluctuations in the raw interferograms appear due to flame flickering, and must be corrected; this has been done using the technique described in [16]. The system was radiometrically calibrated as described in Section 3.

The second stage is the calculation of simulated spectra, using the spectroscopic parameters of the main chemical species of the flame. For a wide range of expected temperatures, the spectral absorptivity α is retrieved from the free online available HITEMP2010 database [7], and its temperature dependence at each wavenumber is parametrized by high-order polynomials [12,15]. Then, emission spectra are calculated with Equation (6) using the Lambert–Beer law to obtain $\tau^{pl}$ as a function of the flame temperature and the column densities. For a hydrocarbon flame that burns all the fuel, the only relevant species are CO${}_{2}$, CO, and H${}_{2}$O, but in the region between 2000 and 2400 cm${}^{-1}$, only CO${}_{2}$ and CO have emission/absorption lines of appreciable intensity. Spectra are also a function of the column density of atmospheric CO${}_{2}$. The emission spectra L(ν) are computed line-by-line in spectral radiance units (W/m${}^{2}\;\cdot \;$sr·cm${}^{-1}$) and are subsequently convolved with the instrumental line shape (ILS) of the IFTS to simulate spectra as measured by the imaging system.

Finally, in the third stage, the simulated and experimental spectra must be compared. An algorithm iteratively changes the parameters used to build the simulated spectrum until an optimal agreement is found with the experimental one. In our case, the parameters are $Q_{CO_{2}}$, $Q_{CO}$, and $T_{pl}$, as well as the atmospheric concentration of carbon dioxide, $Q_{CO_{2}\;atm}$, and the fitting procedure is as follows (a single gas will be assumed in the explanation; for each additional gas the procedure is the same but there is an additional unknown value of column density to be determined). At each pixel, we start by assuming a value for the couple (T${}_{pl}$, Q${}_{pl}$). The theoretical radiance is calculated with Equations (2) and (6) at each point of the wavenumber axis of the experimental spectrum. The differences with the measured radiance spectrum for each wavenumber are added up in quadrature to get the sum of squared errors (SSE). The Nelder–Mead minimization algorithm, as implemented in MATLAB software, is then used to find the value of ($Q_{pl},T_{pl}$) for the next iteration, until convergence is reached. This iterative process is repeated to retrieve values of column density and temperature for each pixel.

**Figure 2:**
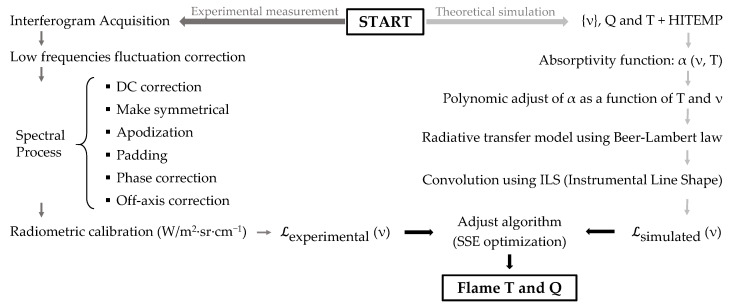
Scheme of the procedure to retrieve temperature and column density from hyperspectral measurements. **Left**: the stage of experimental measurement. **Right**: the stage of construction of simulated spectra.

The complete process is schematized in Figure 2, and it ends up providing images (“maps”) of T and Q for the flame.

Retrieval of compositional data, spatial resolution, and the high accuracy for temperature measurements with a very simple setup, are all very appealing features of the hyperspectral method just described. However, the process of iteratively generating simulated spectra for each pixel until convergence is reached is computationally intensive and may take hours to finish on a high-performance PC. This is a handicap for practical flame temperature retrieval; in particular, the method cannot be used for continuous monitoring. Therefore, a simpler and faster alternative, even if some accuracy in the measured values is lost, would be highly appreciated for industrial applications. This is where multispectral imaging comes in.

### 2.3. Measurement Process: Multispectral

#### 2.3.1. Effect on Spectra of Temperature and Concentration

Measurement by hyperspectral emission spectroscopy, as just described, is based on the dependence on T and Q of the high-resolution spectra of gases, with individual lines resolved. However, lines are grouped into ro-vibrational bands, and when the intensity of the lines changes because of temperature, the overall shape and intensity of the band also change. The reason is that as T increases the emitted radiance in each line increases, but the effect is stronger for lines far from the band center because higher energy rotational levels become more populated, and thus the band becomes wider. Therefore, it is possible, at least in principle, to measure T and Qwith an instrument whose spectral resolution is not fine enough to resolve individual lines.

**Figure 3:**
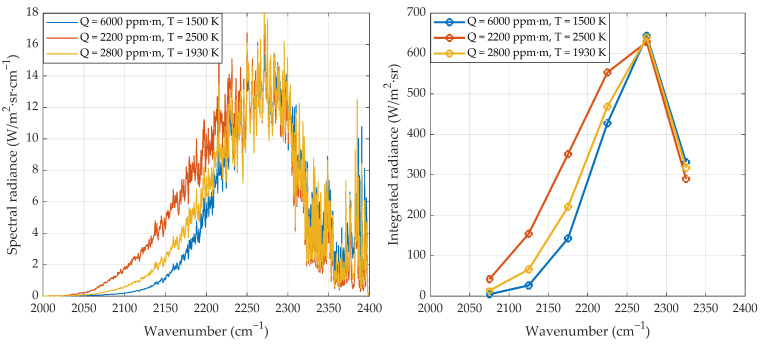
**Left**: Three simulated emission spectra for different column densities Q of CO${}_{2}$ and flame temperatures. **Right**: Integrated radiances over spectral bands of 50/cm ${}^{-1}$ width for the same high−resolution spectra of the left. (Figure previously published in [11]).

This effect can be seen in Figure 3, which shows at the left-hand side calculated spectra (using HITEMP2010 parameters) for three different temperatures and column densities of CO${}_{2}$ (the sharp decrease for ν≳2300 cm${}^{-1}$ is due to absorption by the cold atmospheric CO${}_{2}$). In the right-hand side of the figure these spectra have been integrated over spectral intervals 50 /cm ${}^{-1}$ wide to simulate a multispectral measurement. Values of T and Q have been chosen to give the same spectral radiance at 2275 /cm ${}^{-1}$, but it is clear that the ambiguity can be resolved using the information of additional bands.

This suggests that retrieval of both temperature and species concentration from the multispectral measurement can be made, as in the hyperspectral case, by the iterative fitting of the experimental data with the theoretical simulation, but the simulation will include now an additional step of integration over the spectral band of the respective optical filter.

#### 2.3.2. Calibrated Multispectral Measurements: Definition of Pseudospectra

As in the hyperspectral case, the multispectral process for temperature retrieval can be described in three stages. The first one, the experimental measurement, is performed with an infrared camera in which a set of transmittance filters define a discrete number, *n*, of spectral channels. To radiometrically calibrate the instrument, a nominal spectral width $\Delta_{i}$ is defined for each filter as the full width at half maximum (FWHM) of its spectral transmittance. A blackbody radiator is set at different temperatures and the measured digital number (DN${}_{i}$) is plotted versus the incoming radiance, integrated over $\Delta_{i}$. A least-square linear fitting provides the parameters ${Gain}_{i}$ (slope) and ${Offset}_{i}$ (y-intercept). The experimental radiance in the spectral interval of the *i*-th channel can be subsequently obtained from the measured digital number as

(8) $$L_{i}=\frac{{\mathrm{DN}}_{i}-{Offset}_{i}}{{Gain}_{i}}.$$

Since the camera is calibrated for the incoming radiance, the effect of atmospheric absorption must be taken into account, multiplying the blackbody emitted radiance by the average transmittance over the spectral interval $\Delta_{i}$.

When a multispectral measurement has been made, Equation (8) provides a *measurement vector* ($L_{1},\ldots ,L_{n}$) with the radiometric information. Values of $L_{i}$ do not depend on the transmittance of the filter, since calibration is made against the total integrated radiance over the spectral width $\Delta_{i}$, but they do depend strongly on its spectral width. Therefore, it is convenient to divide $L_{i}$ by $\Delta_{i}$, to have an estimation of the spectral radiance at each filter central wavenumber. The normalized measurement vector $(\frac{L_{1}}{\Delta_{1}},\ldots ,\frac{L_{n}}{\Delta_{n}}$) is the output of the multispectral measurement, and may be considered a low-resolution approximation to the real emission spectrum as measured at the location of the camera; it will be called *pseudospectrum* in this work.

#### 2.3.3. Pre-Calculation of Simulated Pseudospectra

The use of an infrared camera instead of an IFTS fulfills the aim of achieving a cheaper measuring system. The additional aim of a faster and simpler processing needs to be addressed in the following stages. The simulation of the pseudospectra is straightforward: the process is the same as in the hyperspectral case, with the additional step of integration over the spectral intervals $\Delta_{i}$ of the high-resolution spectra. The key to simplifying the method and making it faster is the third stage of the process: comparison between experimental and simulated pseudospectra. The bottleneck of the hyperspectral retrieval process is the iterative generation of simulated spectra to fit the experimental spectrum. Thus, a great efficiency improvement could in principle be achieved by avoiding that process. This can be done if the simulated emission spectra are pre-calculated, as follows:

1. For a specific scene, a set of $\left\{T_{i}\right\}$ and $\left\{Q_{i}\right\}$ values can be defined, such that their ranges cover the expected values in the flame. Nominal emission pseudospectra can be calculated for all the values of the (T, Q) matrix (for a given atmospheric transmittance). A *simulated pseudospectra datacube* is thus obtained;
2. An experimental pseudospectrum can now be compared to all the pseudospectra of this datacube; the ($T_{f},Q_{f}$) couple retrieved is the one that gives the smaller error. The discrepancy between experimental and theoretical pseudospectra was quantified as the sum of absolute errors (SAE), instead of the sum of squared errors (SSE), as used in the hyperspectral method, in order not to excessively weight the errors of a single filter.

Simulated pseudospectra were pre-calculated for two different ranges of T and Q: for the measurements on the standard flame, T was varied between 250 K and 3000 K and Q between 100 ppm·m and 5000 ppm·m; for the measurements on the Bunsen flame, T was varied between 250 K and 2500 K, and Q between 10 ppm·m and 2500 ppm·m. On the basis of the expected errors determined in [11], the steps of ΔT=10 K were used for temperatures, while for column densities the values were ΔQ=20 ppm·m for the standard flame and ΔQ=10 ppm·m for the Bunsen flame.

As the final stage of the process, the experimental pseudospectrum was compared exhaustively, for each pixel, with the full set of the simulated ones to assign it to the (T${}_{f}$, Q${}_{f}$) couple that returned the best match. Exhaustive comparison is not the fastest method to find a minimum error, but computation time is very affordable because of the small size of the data handled, and it ensures the lowest error solution, unlike a search algorithm, which could end up in a local minimum.

## 3. Experimental Setup and Instrumentation

The experimental setup is simply the practical realization of the scheme of Figure 1, with a uniform low reflectance background at room temperature. Two different flames (the standard flame developed at NPL and an ordinary Bunsen flame) and two different imaging instruments (hyperspectral and multispectral) have been used.

**Figure 4:**
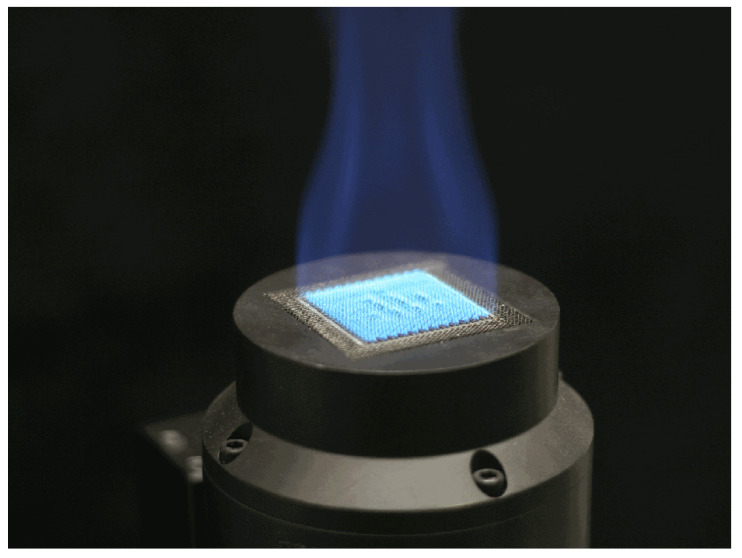
Standard flame burner (Figure previously published in [11]).

The standard flame was developed by NPL in the framework of the EMPRESS [3] project. The burner uses propane as fuel and produces a square array (40 × 40 mm) of small diffusion flamelets stabilized above it, with a zone of nearly uniform temperature and composition (Figure 4). It can be set to different equivalence ratios, from ϕ=0.8 (lean flame) to ϕ=1.4 (rich flame). Temperature, species concentration, and flame dynamics vary with the equivalence ratio. The hottest and more stable flame is the stoichiometric (ϕ=1), which has also a very small amount of CO [3]. An additional feature of the standard flame is that the spatial profiles of temperature and species concentration are very flat, and therefore the assumption of uniform temperature and concentration along the line of sight is fully justified.

Measurements were also performed in a Bunsen burner using butane as fuel (model Labogaz 206 from Campingaz) to test the multispectral method in an ordinary flame similar to those used in many industrial applications. The equivalence ratio could not be measured, but the fuel inlet was regulated to obtain a flame approximately stoichiometric.

To ensure stability, both flames were turned on for one hour before the measurements were performed, and the doors and windows of the room remained closed throughout the process to keep temperature stable and to avoid drafts that could cause movements in the flame.

The IFTS used in this work is an FTIR hyperspectral Imaging System (Telops FIRST-MW) operating in the extended mid-infrared (MIR) region, from 1850 /cm to 6600 /cm. The system consists of a Michelson interferometer coupled to an InSb focal plane array (FPA), with 320 × 256 pixel resolution, an instantaneous (pixel) field of view of 0.35 mrad, and a maximum resolution of 0.25 /cm${}^{-1}$. In order to reduce the long acquisition time to ∼2 or 3 min, all spectra have been measured at 0.5 /cm${}^{-1}$ in a sub-window of 160 × 256 pixels. More information about this system can be found in [12].

The IFTS was radiometrically calibrated at Centro Español de Metrología (CEM). Two blackbodies were used with an aperture large enough (70 mm diameter) to cover the whole field of view of the sub window used. Calibration temperatures were 180 ${}^{\circ }$C and 400 ${}^{\circ }$C. Since the spectral emission of the flame is concentrated in a narrow band in comparison to the full spectral range of the instrument, these blackbody temperatures were sufficient to calibrate the instrument for flame temperatures up to 2500 ${}^{\circ }$C [3,17].

**Figure 5:**
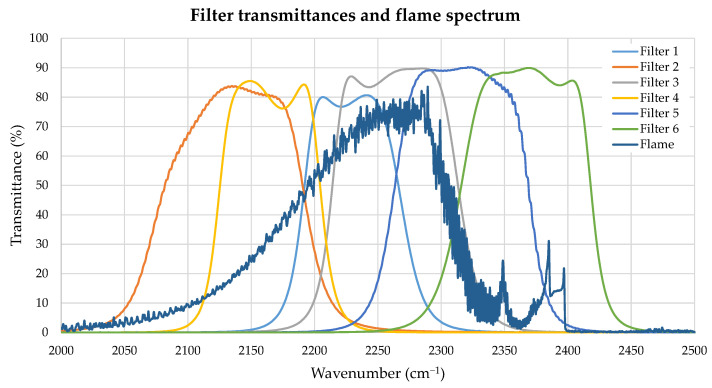
Transmittance in the mid−IR region of the six interference filters used, with a emission spectrum of a the standard flame (in arbitrary units) shown for comparison.

On the other hand, the multispectral measurements were performed using a Thermosensorik SME 640 camera, that operates in the MIR band (3 to 5 μm), with a 640 × 512 InSb Stirling-cooled FPA detector. It features a rotating wheel placed immediately after the optics, with a capacity for six interference filters of 1-inch diameter. The spectral transmittance profiles of the filters used are shown in Figure 5, superimposed to a typical emission spectrum of the standard flame (in arbitrary units). For each filter, a different integration time was used in order not to saturate the camera response, and a radiometric calibration was performed with a 15 × 15 cm extended area blackbody radiator (model 4006 G from Santa Barbara Infrared, Inc., Santa Barbara, CA, USA) whose emissivity had been previously measured at Centro Español de Metrología (CEM).

In all multispectral measurements, the effect of atmospheric transmittance was taken into account using a standard atmospheric CO${}_{2}$ meter and calculating the corresponding value for the atmospheric path for calibration and for measurement. In hyperspectral measurements, the concentration of atmospheric CO${}_{2}$ was calculated by iterative fitting, with results in good agreement with those of the CO${}_{2}$ meter.

## 4. Experimental Results: Six Filters

Since in previous works hyperspectral temperature measurements in the NPL standard flame have proven very accurate, as explained in the Introduction, we will assume throughout this article that the results of the hyperspectral method are our “ground truth”’, and use them to validate the multispectral temperature maps. Multispectral column density will be compared also with the hyperspectral value, although in this case it should be considered simply as a reference rather than a “ground truth”’ since there is no independent experimental validation of its results.

### 4.1. Measurements with Six Filters: Standard Flame

**Figure 6:**
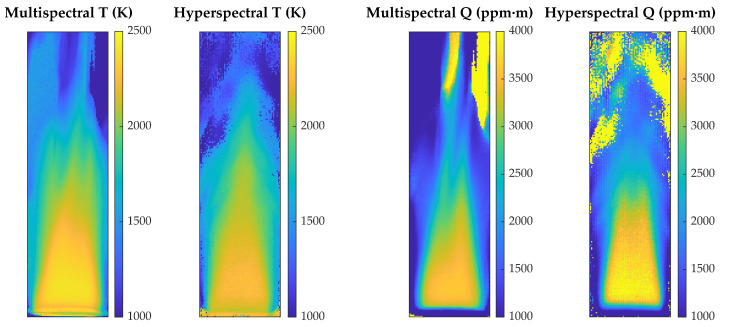
Maps of temperature and CO${}_{2}$ column density for the stoichiometric (ϕ=1) standard flame, obtained with the multispectral method and with the hyperspectral method.

Figure 6 provides a visual comparison between multispectral and hyperspectral results for the standard flame in the stoichiometric case (ϕ=1) . Values are qualitatively very similar in the flame region, although they show important differences where the gases are colder. This is to be expected since results are unreliable outside the central zone, because radiance, and consequently signal-to-noise ratio (SNR), decrease sharply outside of the burner area, which translates into erroneous estimates of T and Q. However, multispectral results seem to be more robust in areas of low radiance, where the hyperspectral maps have more pixels with obviously wrong values.

On the other hand, since the value of radiance in a specific spectral region can be kept constant by increasing T and decreasing Q or vice versa, regions or low SNR are prone to errors in which a T value too high corresponds to a Q value too low, or vice versa. This can be seen very clearly in the areas at the top of Figure 6, where the pixels with temperature values at the bottom of the scale have column density values at the top of the scale.

**Figure 7:**
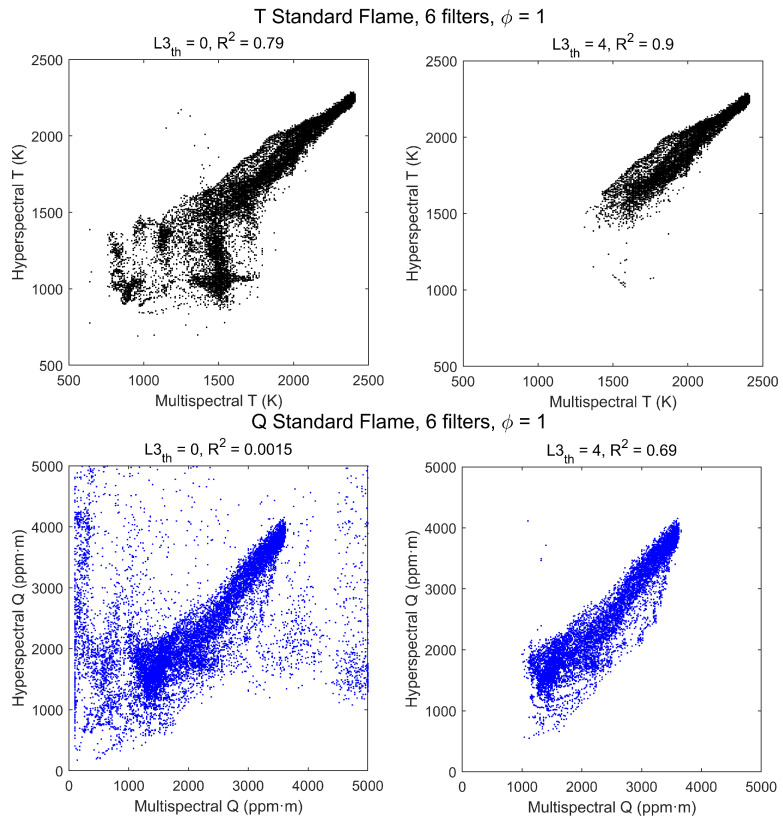
Scatterplots of hyperspectral vs. multispectral retrieved values of T (**top row**) and Q (**bottom row**) for the stoichiometric (ϕ=1) standard flame. Each point corresponds to a pixel, with no radiance threshold (**left column**) and with threshold L3${}_{th}=4$ W/m${}^{2}\;\cdot \;$sr·cm${}^{-1}$ (**right column**).

A more detailed comparison of results can be made by plotting the hyperspectral temperatures and CO${}_{2}$ column densities versus the multispectral values in a scatterplot, as in Figure 7, where each dot corresponds to a pixel of the image. In the plots on the left-hand side, all pixels of Figure 6 are plotted, and values are very noisy for regions of small signal. However, if the plot is restricted to pixels from regions of larger radiance the correlation becomes much better. The effect of radiance level has been studied by including only pixels above a variable radiance threshold. To be specific, the third element of the pseudospectra has been used, $L3\equiv \frac{L_{3}}{\Delta_{3}}$. Setting this threshold to a spectral radiance of $L3_{th}=4$ W/m${}^{2}\;\cdot \;$sr·cm${}^{-1}$ gives the scatterplots at the right-hand side of Figure 7. Now the correlation is much better, as indicated by the $R^{2}$ values shown in the graph, and it can be improved further, especially for Q, by increasing the L3 threshold; for instance, if $L3_{th}=6$ W/m${}^{2}\;\cdot \;$sr·cm${}^{-1}$ it is found that $R^{2}\left(Q\right)=0.85$. This shows that, with enough radiance signal, nearly all T and Q information provided by the hyperspectral method is captured by the multispectral measurements.

**Figure 8:**
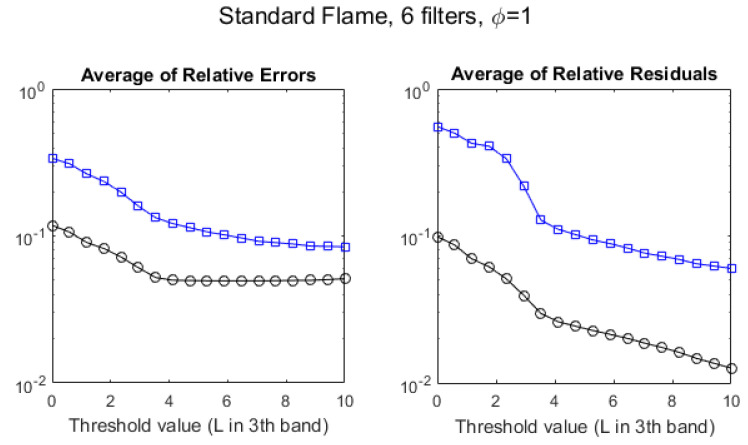
Evolution of the discrepancies between hyperspectral and multispectral temperatures (black circles) and column densities (blue squares), measured as average relative error (**left**) and average residuals (**right**), for the stoichiometric (ϕ=1) standard flame, as a function of the radiance threshold, measured in W/m${}^{2}\;\cdot \;$sr·cm${}^{-1}$.

The agreement between temperatures measured by the hyperspectral method, $T_{i}^{H}$ and by the multispectral method, $T_{i}^{M}$, can be quantified by the *average of relative errors* (ARE) of the $T_{i}^{M}$ values, defined as ARE $\equiv \langle |T_{i}^{H}-T_{i}^{M}|/T_{i}^{H}\rangle$ (and, in an analogous way, for $Q_{i}^{M}$). The effect of increasing the radiance threshold can be seen, for T and Q, at the left-hand side of Figure 8. The ARE for the multispectral T (black circles) stabilizes in ∼5% for pixels above $L3_{th}=4$ W/m${}^{2}\;\cdot \;$sr·cm${}^{-1}$, whereas for the multispectral Q (blue squares) it is ∼10% and keeps decreasing slightly for larger thresholds.

In fact, since there is a good correlation between both methods, in particular for temperature, the linear regression of the hyperspectral values on the multispectral values can be used to improve the estimations. If the slope and y-intercept of that regression are *m* and *b*, respectively, the estimated hyperspectral temperatures, based on the multispectral measurements are ${\hat{T}}_{i}^{H}=m\;\cdot \;T_{i}^{M}+b$, and the residuals are $T_{i}^{H}-{\hat{T}}_{i}^{H}$. The *average of the relative residuals*, defined by ARR $\equiv \langle |T_{i}^{H}-{\hat{T}}_{i}^{H}|/T_{i}^{H}\rangle$ is a meaningful benchmark of the accuracy of the multispectral measurement of temperature (in an entirely analogous way, it can be defined for Q). The right-hand side of Figure 8 plots this parameter for T and Q as a function of the radiance $L3_{th}$ threshold. It can be seen that estimating the temperatures using the regression line may reduce it to values as low as nearly ∼1% if only pixels with large radiances are considered.

It is worth pointing out that, as observed in Figure 7, for high temperatures, hyperspectral values tend to be below multispectral values, whereas the opposite is true for column densities. This seems to be another example of the relative equivalence for radiance levels of lowering T and increasing Q, as mentioned before with respect to errors in the T and Q maps. Using the linear regression to estimate T and Q corrects this effect, and therefore relative errors of the residuals of T and Q should be smaller than those of the multispectral values, as observed.

**Figure 9:**
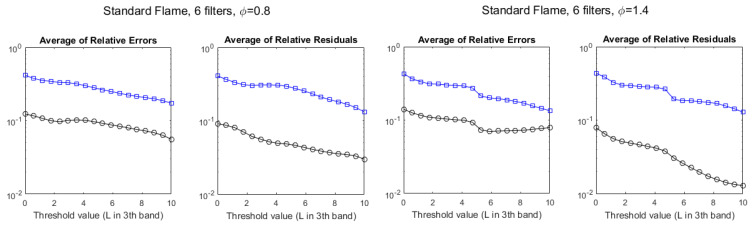
Evolution of the discrepancies between hyperspectral and multispectral temperatures (black circles) and column densities (blue squares), measured as average relative error and average residuals, for the standard flame, as a function of the radiance threshold, measured in W/m${}^{2}\;\cdot \;$sr·cm${}^{-1}$. **Left**: lean flame (ϕ=0.8). **Right**: rich flame (ϕ=1.4).

The same study has been conducted for the rich flame (ϕ=1.4) and the lean flame (ϕ=0.8), with similar results, although the agreement with hyperspectral values is somewhat worse, especially for column densities (see Figure 9).

### 4.2. Measurements with Six Filters: Bunsen Flame

The flame of the Bunsen burner has several important differences to the standard flame: radiance levels are smaller, the flame is less stable, and the temperature and composition of the flame are spatially inhomogeneous along the line of sight. The aim of the measurements in this section is to study whether these factors, which in principle make multispectral measurements less reliable, affect the retrieved values of T and Q.

**Figure 10:**
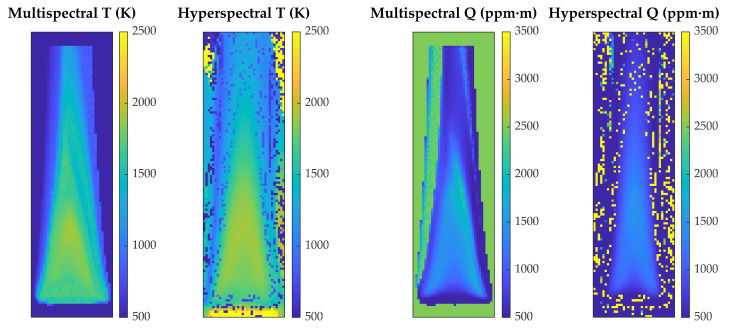
Maps of temperature and CO${}_{2}$ column density for a Bunsen flame, obtained with the multispectral method and with the hyperspectral method.

Figure 10 compares the temperature and column density maps obtained by the hyperspectral and multispectral methods. Again, values are qualitatively very similar in the flame region, but now there are many pixels with anomalous values of T and Q in regions closer to the main flame. This is in fact to be expected, since radiance levels in the Bunsen flame are smaller than in the standard flame, because temperatures are lower but also because the flame is thinner, and column densities are considerably smaller. As in the standard flame, most of the anomalous values correspond to Q too high and T too low, but there are also pixels with the opposite behavior at the top and the bottom of the image (yellow in the hyperspectral Q map). Almost all of these pixels appear in the hyperspectral images, showing again that the multispectral method is more robust in regions of low signal.

**Figure 11:**
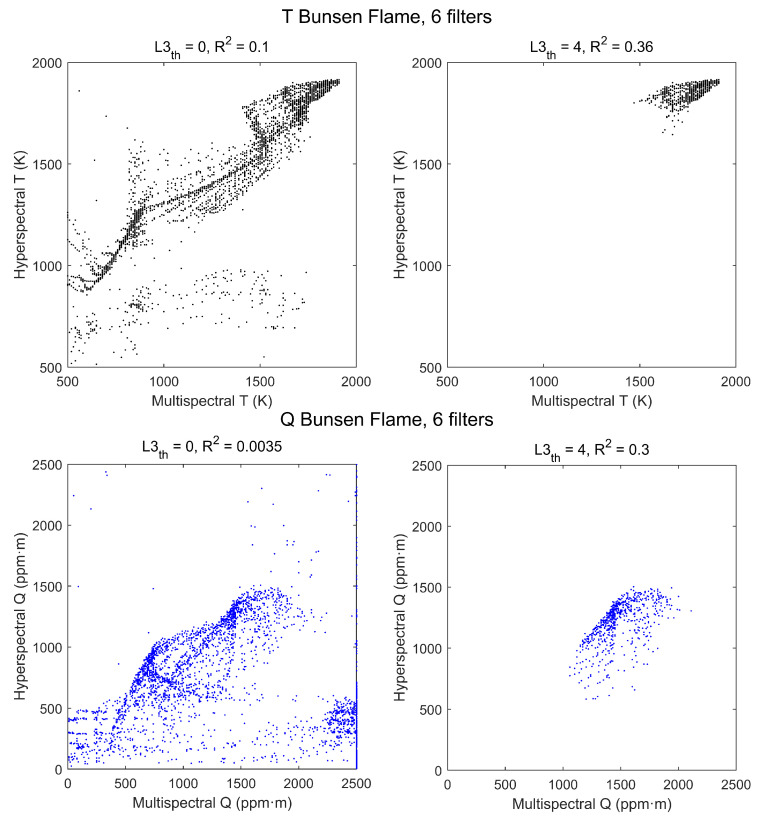
Scatterplots of hyperspectral vs. multispectral retrieved values of T (**top row**) and Q (**bottom row**) for a Bunsen flame. Each point corresponds to a pixel, with no radiance threshold (**left column**) and with threshold L3${}_{th}=4$ W/m${}^{2}\;\cdot \;$sr·cm${}^{-1}$ (**right column**).

The effect of the radiance threshold on the scatterplots is apparent in Figure 11. Values of $R^{2}$ are considerably worse than those of the standard flame, even for $L3_{th}=4$ W/m${}^{2}\;\cdot \;$sr·cm${}^{-1}$, but these data are somewhat deceptive because with that threshold, due to the lower level of radiance, only a small region of the flame is selected and the range of T and Q values is too small to show a good correlation. The smaller values of Q explain also why the threshold used selects now temperatures above ∼1650 K instead of ∼1350 K as in the standard flame.

**Figure 12:**
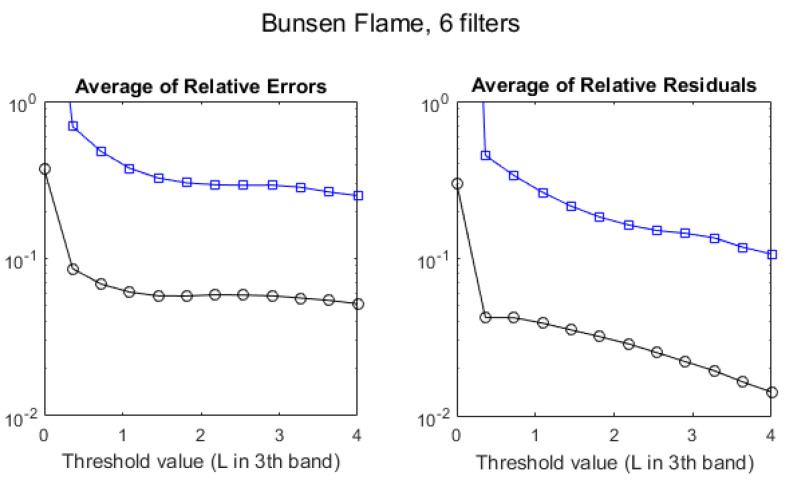
Evolution of the discrepancies between hyperspectral and multispectral temperatures (black circles) and column densities (blue squares) for a Bunsen flame, measured as average relative error (**left**) and average residuals (**right**), as a function of the radiance threshold, measured in W/m${}^{2}\;\cdot \;$sr·cm${}^{-1}$.

Despite the poor values of $R^{2}$, the error benchmarks, plotted in Figure 12, exhibit a behavior similar to that of the standard flame, showing that the multispectral method is also applicable to this flame with a similar level of accuracy.

## 5. Experimental Results: Reduced Number of Filters

In what we have discussed so far, the radiance of the six filters of the multispectral system has been used to retrieve temperature and column density values. This is the natural procedure, since, in principle, the more spectral information we have, the better our results will be. However, there are several reasons why it could be advantageous to base the retrieval on a smaller number of bands; for instance, to avoid spectral regions with larger uncertainty (e.g., those more influenced by the presence of atmospheric CO${}_{2}$), or to discard bands that may have co-registration errors with the others, due to lack of flame stability, or bands that could be saturated or close to saturation. And of course, the possibility of a system that uses fewer bands is of interest in itself, because it would be cheaper and more simple.

### 5.1. Measurements with a Reduced Number of Filters: Standard Flame

**Figure 13:**
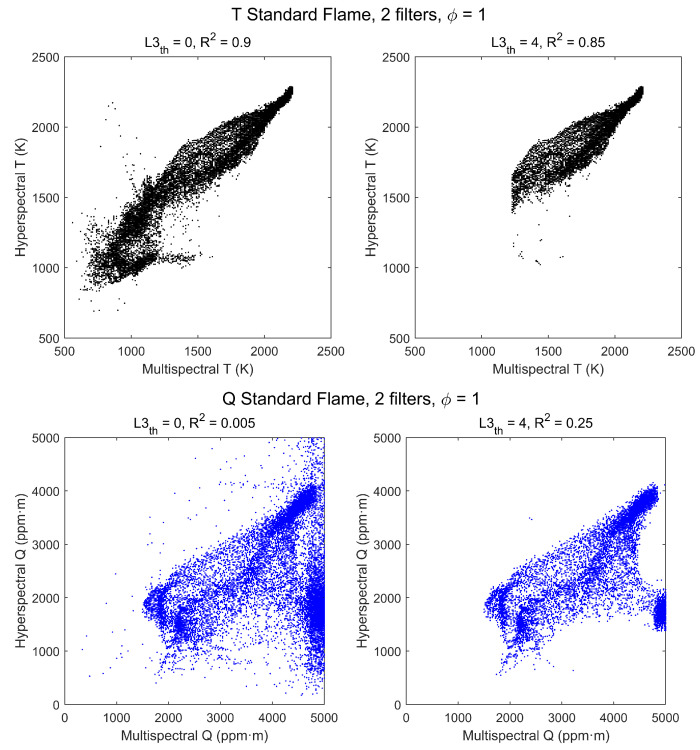
Scatterplots of hyperspectral vs. multispectral retrieved values of T (**top row**) and Q (**bottom row**) for the stoichiometric (ϕ=1) standard flame, using only filters 1 and 3 for retrieval. Each point corresponds to a pixel, with no threshold radiance (**left column**) and with threshold L3${}_{th}=4$ W/m${}^{2}\;\cdot \;$sr·cm${}^{-1}$ (**right column**).

Two possibilities have been studied: using the first four filters (thus discarding information from filters 5 and 6, the most affected by atmospheric CO${}_{2}$, see Figure 5) and using only filters 1 and 3 (corresponding to the regions with larger signal and no contribution from CO). The retrieved temperature and column density maps, in all cases, have a visual appearance similar to those obtained with the six filters. Hyperspectral vs. multispectral scatterplots are also very similar for six and four filters, showing that discarding filters 5 and 6 does not cause a significant loss of information. However, appreciable differences appear in the case of two filters. This can be seen for the standard flame in stoichiometric conditions by comparing Figure 13 with Figure 7: temperatures retrieved with two filters tend to be somewhat lower than hyperspectral temperatures (instead of somewhat larger, as temperatures retrieved with six filters), and the $L3_{th}$ threshold causes now a sharp threshold in temperatures; however, correlation is similar, and even better for the zero-threshold case. In contrast, the correlation for column densities is much worse, and for the zero-threshold case there is a large cluster of pixels with Q values near the top of the range, that is, 5000 ppm·m. These values are clearly wrong but could probably assume better values if the range of Q for the pre-calculated would be increased.

**Figure 14:**
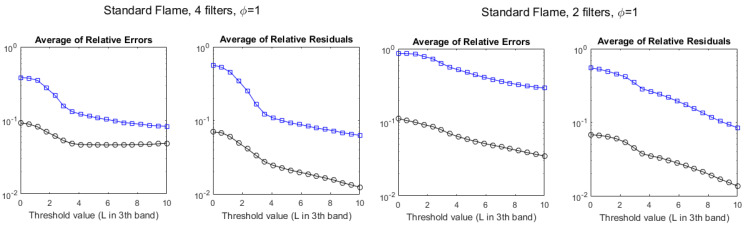
Evolution of the discrepancies between hyperspectral and multispectral temperatures (black circles) and column densities (blue squares), measured as average relative error and average residuals, for the standard stoichiometric flame, as a function of the radiance threshold, measured in W/m${}^{2}\;\cdot \;$sr·cm${}^{-1}$. **Left**: values retrieved with four filters. **Right**: values retrieved with two filters.

The average of relative errors and the average of relative residuals, as a function of the threshold for the stoichiometric standard flame, can be seen in Figure 14. The comparison with results for six filters, in Figure 8, shows nearly no difference when using four filters. Using two filters, the results for Q are worse, but the results for T are similar if measured by the ARR, and even slightly better if measured by the ARE, at least for the large values of $L3_{th}$. Similar behavior is found for the non-stoichiometric cases in the standard flame (not shown).

### 5.2. Measurements with a Reduced Number of Filters: Bunsen Burner

**Figure 15:**
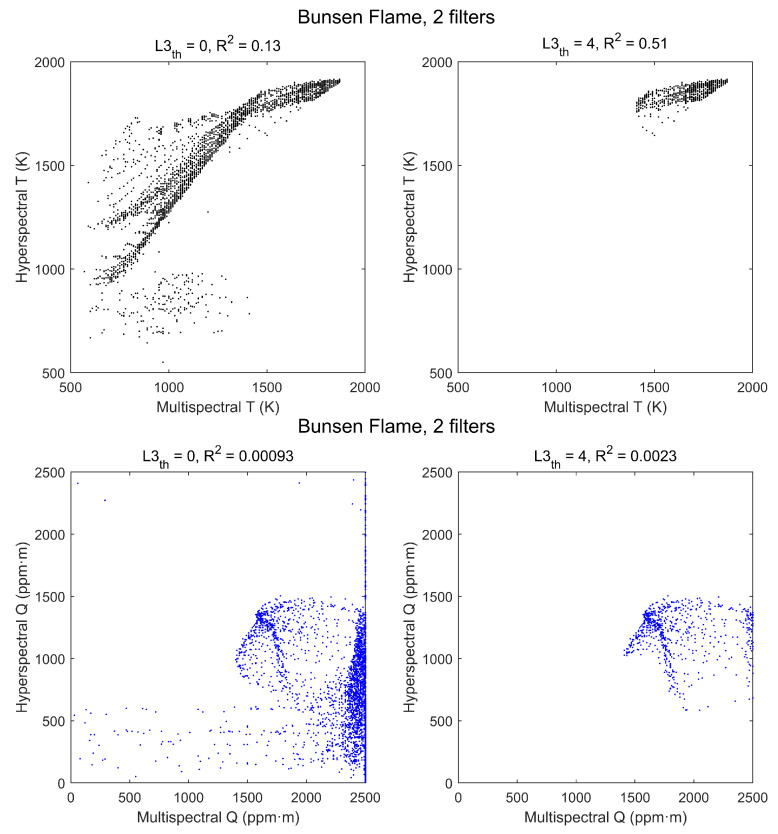
Scatterplots of hyperspectral vs. multispectral retrieved values of T (**top row**) and Q (**bottom row**) for the Bunsen flame, using only filters 1 and 3 for retrieval. Each point corresponds to a pixel, with no radiance threshold (**left column**) and with threshold L3${}_{th}=4$ W/m${}^{2}\;\cdot \;$sr·cm${}^{-1}$ (**right column**).

The effects of reducing the number of spectral channels in the Bunsen flame measurements are similar to those already exposed for the standard flame. The main differences appear when only two filters are used, as shown in Figure 15. With the radiance threshold $L3_{th}$ temperatures are hardly affected, whereas correlation for column densities is much worse. With no radiance threshold, column densities show a large cluster of pixels with obviously wrong values near the top of the range of pre-calculated spectra. This is the same effect found in the standard flame, although here the top of the range is Q = 2500 ppm·m.

**Figure 16:**
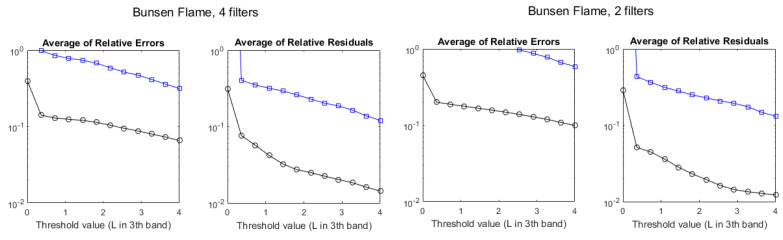
Evolution of the discrepancies between hyperspectral and multispectral temperatures (black circles) and column densities (blue squares), measured as average relative error and average residuals, for the Bunsen flame, as a function of the radiance threshold, measured in W/m${}^{2}\;\cdot \;$sr·cm${}^{-1}$. **Left**: values retrieved with four filters. **Right**: values retrieved with two filters.

The averages of relative errors and relative residuals using four filters (Figure 16) have also values very similar to those found using six filters (Figure 12). Using only two filters causes a large degradation of the ARE for Q and a small one for T, whereas changes in the ARR are small.

## 6. Summary, Conclusions & Future Work

Hyperspectral imaging in the mid-IR is capable of providing spatially resolved accurate measurements of flame temperature (T), as well as column densities (Q) for the main chemical species present, but it has the disadvantage of requiring expensive instrumentation and complex processing. In this work, these two problems have been approached by using a multispectral system built with a camera that operates in the mid-IR and has six channels defined by interference filters, and by proposing a fast method of retrieval of T and Q${}_{{CO}_{2}}$ based on the pre-calculation of emission spectra, simulated line-by-line using the HITEMP2010 spectroscopic database, which are compared to the experimental multispectral measurements. This approach overcomes the difficulty that unknown emissivity poses to thermography of flames, because simulation of spectra effectively parametrizes the spectral emissivity of the flames as a function of T and Q${}_{{CO}_{2}}$.

The results have been systematically compared with those of the hyperspectral method for a standard flame of well-known composition and temperature, studied in previous works, and for an ordinary Bunsen flame. In each case, two benchmarks have been calculated to quantify the agreement of the multispectral temperatures and column densities with the hyperspectral values: the average of relative errors (ARE) and the average of relative residuals (ARR) of the values estimated by means of a linear regression.

The degree of agreement has been found to depend strongly on the level of radiometric signal of the flame, but for levels of radiance that correspond to T ≳1300 K in the standard flame, ARE in temperature is ∼5%, whereas the ARR is ∼2.5% and can be as low as nearly ∼1% for larger levels of radiance. Results in the Bunsen flame are comparable, although overall radiance levels are smaller, because maximum temperatures and, especially, column densities have lower values.

For all the cases studied it has been found that agreement with the hyperspectral results is much better for temperatures than for column densities. This is to be expected, since measurement in emission mode is always more sensitive to temperatures than to concentrations. An unexpected result, however, has been that the multispectral method has proven more robust than the hyperspectral method, with fewer pixels with obviously wrong values in the regions of low radiance levels.

One of the main findings of this work is that values of T retrieved with only four, or even two filters, are nearly as accurate as those obtained with the full multispectral information of the six filters. Values of Q, on the other hand, are not affected by using four filters instead of six, but are clearly worse when using only two. The feasibility of accurate bi-spectral thermometry in flames is a very promising result, because a bi-spectral system could be implemented without the need for moving parts using two detectors with independent optics, or possibly dual-band detectors. The ability to measure temperature and CO${}_{2}$ column density with only two spectral channels opens also the possibility to use other channels to measure other chemical species; in particular, CO and unburned hydrocarbons, which have emission lines in the mid-IR. This can be very useful to estimate combustion efficiency, a critical parameter in many industrial applications.

A critical factor that has limited the performance of both the multispectral and the hyperspectral methods has been flame stability, in particular for the less energetic regions, that are also more easily affected by air drafts. The regular flame flickering can be dealt with, thanks to the specific processing developed in [16], but since hyperspectral measurements take typically a few minutes, it is essential that the flame does not change its dynamics during that period of time. An advantage of multispectral measurements is that they can be much faster, but in our case they have taken a similar time because the filter wheel position and the integration time were changed manually. There is thus room for improvement by automating this process; this could reduce the measurement time to ∼1 s or even less and would increase accuracy for regions outside the flame core.
